# Polymer‐Coated Metal‐Oxide Nanoparticles Inhibit IgE Receptor Binding, Cellular Signaling, and Degranulation in a Mast Cell‐like Cell Line

**DOI:** 10.1002/advs.201500104

**Published:** 2015-07-14

**Authors:** Van A. Ortega, James D. Ede, David Boyle, James L. Stafford, Greg G. Goss

**Affiliations:** ^1^ Department of Biological SciencesUniversity of AlbertaEdmontonAlbertaCanadaT6G 2E9; ^2^ National Research Council (Canada)National Institute for NanotechnologyEdmontonAlbertaCanadaT6G 2M9

**Keywords:** cytotoxicity, immunology, nanoparticles, receptors, viability

## Abstract

Previous reports have shown that nanoparticles (NPs) can both enhance and suppress immune effector functions; however the mechanisms that dictate these responses are still unclear. Here, the effects of polyacrylic acid (PAA) functionalized metal‐oxide NP are investigated on RBL‐2H3 (representative mammalian granulocyte‐like cell line) cell viability, cellular degranulation, immunoglobulin E (IgE) receptor binding, and cell signaling pathways related to immune function. The increasing development of PAA‐NPs as pesticide dispersants and as drug carriers in therapeutics necessitates their investigation for safe production. Using two in vitro experimental approaches, this study demonstrates that pre‐exposing RBL‐2H3 cells, or IgE antibodies, to PAA‐NPs (TiO_2_, CeO_2_, ZnO, Fe_2_O_3_, and PAA‐Capsules (NP coating control) over 24 h, significantly decrease the binding capacity of IgE for Fcε receptors, inhibit the phosphorylation of intracellular signaling proteins (e.g., MAPK ERK) that mediate degranulation, and inhibited RBL‐2H3 cell degranulation. In addition, and unlike the other NPs tested, PAA‐TiO_2_ significantly reduced RBL‐2H3 viability, in a time (4–24 h) and dose‐dependent manner (>50 μg mL^−1^). Together, these data demonstrate that PAA‐NPs at sub‐lethal doses can interact with cell surface structures, such as receptors, to suppress various stages of the RBL‐2H3 degranulatory response to external stimuli, and modify immune cell functions that can impact host‐immunity.

## 1 Introduction

The unique physico‐chemical properties of nanoparticles (NPs) have captured the interest of the consumer industry and the biomedical community for their potential to improve product quality and augment practices for the treatment of disease, respectively. When compared to their larger bulk equivalents, materials in the nanoscale have increased charge, larger surface area to volume ratios and can exhibit quantum fluorescence.^1^ As such, NPs are being developed as fluorescent agents for cell imaging, as cancer therapeutics^2^ and as additives in paints and cosmetics, and for use in industrial applications.^1^

This increased use has spurred the rapid development of NPs of varying sizes, material types, and surface functionalizations, each with their own unique characteristics and potential for toxicity (e.g., apoptosis,^3^ cell stress,^4^ and altering catalytic enzymes.^5^, ^6^, ^7^). For example, bare metal NPs aggregate readily, precipitate in solution and often release free metal ions, which can increase the variability of observed effects and obscure the source of NP toxicity.^8^ By contrast, functionalizing metal NPs with polymers such as polyacrylic acid (PAA), increases dispersiveness in solutions^9^ and reduces dissolution of the metal core.^10^ Accordingly, PAA‐NP‐mediated toxicity could be driven by different mechanisms when compared to core materials alone. The need to study PAA‐NP toxicity is particularly relevant given the increasing development of these particles as both pesticide dispersants for the agricultural industry,^11^ and as drug delivery carriers in nano‐medicines.^12^ The common hypothesis is that NPs with identical coatings will interact with biological tissues in a similar manner since the coating will dominate the interaction and potentially lessen toxic effects.^3^ However, the use of highly monodispersed NPs may also alter translocation and deposition across tissues and interactions with cells, when compared to effects from bare NPs. Thus, the safe administration of NPs requires a comprehensive assessment of their biocompatibility with both target and non‐target tissues.

One key consideration in the development of NP‐enabled products is the response of the host immune system to exposure. Indeed, while some NPs have been specifically designed to activate the immune response (e.g., vaccine adjuvants^13^), others have also been shown to either inadvertently suppress^14^ or activate^15^ various immune responses. For example, in rats, intravenous administration of polymeric NPs resulted in decreased serum histamine levels,^14^ while intraperitoneal administration of bare TiO_2_ NPs promoted allergic sensitization and increased the number of inflammatory cells in mice.^15^ However, the mechanisms by which NPs modulate immune responses, and the wider repercussions for immune functioning, are not well established.

**Figure 1:**
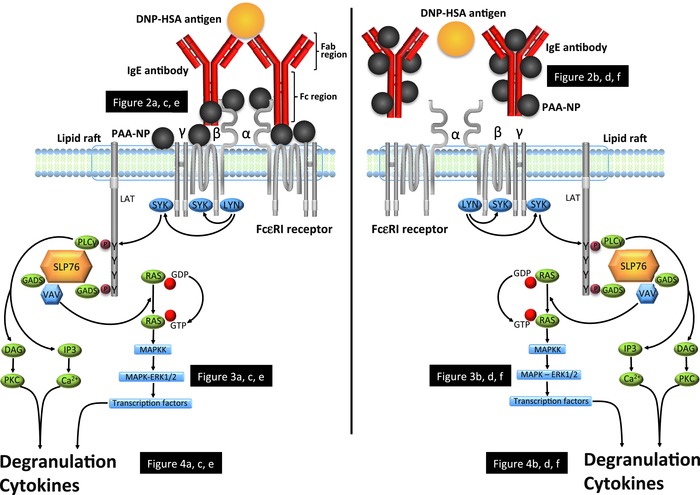
Schematic representation of the degranulatory signaling cascade of RBL‐2H3 (adapted from refs.^17^ and^34^). Degranulation occurs when FcεRI are cross‐linked by antigen‐bound IgE, resulting in the recruitment of tyrosine kinases, *tyrosine‐protein kinase* (LYN) and *spleen tyrosine kinase* (SYK) to the transmembrane *immunoreceptor tyrosine‐based activation motif* (ITAMs) of the receptor. This results in the phosphorylation of tyrosines within the transmembrane adaptor molecule *Linker for Activation of T cells* (LAT) that coordinates the downstream signaling of multiple cytosolic adaptor molecules, including, *GRB2‐related adaptor protein* (GADS), *SH2‐domain‐containing leukocyte protein of 76 kDa* (SLP76), among others not shown for simplicity. As well, the exchange factor, VAV and the signaling enzyme, *Phospholipase Cγ1* (PLCγ), which regulates calcium mobilization and activation of *Protein Kinase C* (PKC), mediate the exocytosis of degranulatory products, such as β‐hexosaminidase. VAV also activates the RAS‐MAPK (ERK) pathway, which stimulates the production of transcription factors that generate cytokines and contributes to degranulation. Two experiments were designed to test the effects of NP exposures on various components of the degranulatory pathway. Experiment 1 was designed to test the effects of NP‐exposed RBL‐2H3 cells degranulation by measuring: 1. IgE binding FcεRI (Figure 2a,c,e), 2. MAPK ERK phosphorylation (Figure 3a,c,e), and 3. Exocytosis of degranulatory products (i.e., β‐hexosaminidase) (Figure 4a,c,e). Experiment 2 tested the effects of NP‐exposed IgE antibodies on identical endpoints of the degranulatory pathway as were measured in Experiment 1 (i.e., Figures 2, 3, 4b,d,f).

Assessing the effects of NPs on innate immunity is particularly important because innate immune cells form the first line of cellular defense against invading pathogens, and as such, are also one of the first immune cell types that are likely to encounter and respond to NPs.^14^ Mast cells are innate cells located throughout the body but especially in mucosal linings of the respiratory and gastrointestinal tract where they act as early detectors and responders to antigenic particles.^16^ Mast cells detect foreign invaders using high affinity surface membrane Fcε receptors (FcεRI), which, when engaged and oligomerized by antigen‐bound immunoglobulin E (IgE) antibodies, mediate cell activation. This oligomerization activates a complex cascade of intracellular signals that terminates in the extracellular release of vesicle‐stored antimicrobial and inflammatory granules (e.g., β‐hexosaminidase (β‐hex), histamines, and others) in a process known as degranulation (see review by Gilfillan and Tkaczyk^17^ for details). In short, the extracellular region of the FcεRI α alpha chain binds the Fc region of IgE, while the Fab region of IgE contains the antigen. Once two FcεRI are cross‐linked, membrane‐bound and receptor‐associated Src‐family kinases (e.g., LYN, SYK) initiate signal transduction by shifting FcεRI to a phosphorylated state, by adding phosphate groups to the tyrosine residues of the *immunoreceptor tyrosine‐based activation motif* (ITAM) on the intracellular β and γ‐chain of the FcεRI tail. Signal propagation is further enhanced by downstream activation of calcium channels and other signal kinases, such as mitogen‐activated protein kinases (MAPK) and related extracellular signal‐related kinases (ERK), to mediate degranulation of the cell (see **Figure** **1**). The immortalized rat basophilic leukemia (RBL‐2H3) cell line is commonly used as a model for in vitro studies of mast cell‐like immune functions,^18^, ^19^ including degranulation. RBL cells express endogenous FcεRI and effectively bind IgE on their surface in a process called sensitization. Once sensitized, RBL‐2H3 can be activated by dinitrophenyl (DNP)‐human serum albumin (HSA), a ligand that stimulates RBL‐2H3 degranulation by cross‐linking IgE‐primed FcεRI.^17^

The propensity for NPs to bind and alter the conformational structure and biological function of proteins,^5^, ^7^ suggests that they may also alter the function of surface membrane receptors, including FcεRIs. Huang et al.^20^ showed that DNP‐albumin labeled gold NPs directly stimulated FcεRI‐mediated responses by providing cross‐linking of receptors. However, bare gold particles themselves did not bind to FcεRI, nor did they alter degranulatory behavior. Intracellular signaling (e.g., MAPK pathway) has been demonstrated to be an effective means to monitor FcεRI‐mediated interactions and cellular effector function in RBL‐2H3 cells.^21^ Thus, the ligand‐receptor‐signal transduction axis can be used to understand how NPs alter immune function.

In this study, we investigated the impacts of various commercially available PAA functionalized metal‐oxide NPs (TiO_2_, ZnO, CeO_2_, and Fe_2_O_3_) on the degranulatory response of RBL‐2H3 cells. Specifically, our aim was to assess the impact of PAA‐NPs on the interaction between FcεRI and IgE, and on activated intracellular signals that determine cell effector functions. A further aim was to provide comparative data on cell function and viability for cells exposed to NPs with distinct metal cores, but possessing identical primary particle size and PAA functionalization. This allows the testing of our underlying hypothesis that similar sized core NPs, functionalized with a common coating, will provide a similar cellular response.

## 2 Results

### 2.1 Characterization of PAA‐NP Spectral Optics

The optical properties were measured for each PAA‐NP to characterize their intrinsic absorbance and fluorescence. Absorbance for each PAA‐NP occurred at around 250 nm, with PAA‐TiO_2_ absorbing the highest at nearly 1.0 a.u. (Supplementary Figure 1a, Supporting Information). None of the PAA‐NPs displayed fluorescent properties, aside from PAA‐Fe_2_O_3_, which emitted fluorescence (90 RFU) at around 350 nm, when excited at 250 nm (Supplementary Figure 1b, Supporting Information).

### 2.2 RBL‐2H3 Viability was Reduced when Cells were Pre‐exposed to PAA‐TiO_2_

After 4 h, RBL‐2H3 viability was significantly decreased to 88 ± 2.1% of control (100 ± 1.0%), when exposed to 200 μg mL^−1^ PAA‐TiO_2_, which was further decreased to 67 ± 8.8% of control (100.3 ± 0.4%) after 24 h (Supplementary Figure 2a, Supporting Information). At this same time point, viability was also reduced to 87 ± 5.5% from exposures to 100 μg mL^−1^ PAA‐TiO_2_. In contrast, exposure to PAA‐ZnO, PAA‐Fe_2_O_3_, PAA‐CeO_2_, and PAA‐Cap NPs had no significant effects on cell viability at concentrations ≤200 μg mL^−1^ and for exposures up to 24 h (Supplementary Figure 2b–e, Supporting Information). No changes in viability were observed for concentrations less than 50 μg mL^−1^ over 24 h for any of the PAA‐NPs (data not shown).

### 2.3 PAA‐TiO_2_‐Exposed RBL‐2H3 Cells had Reduced Binding of IgE to FcεRI

**Figure 2:**
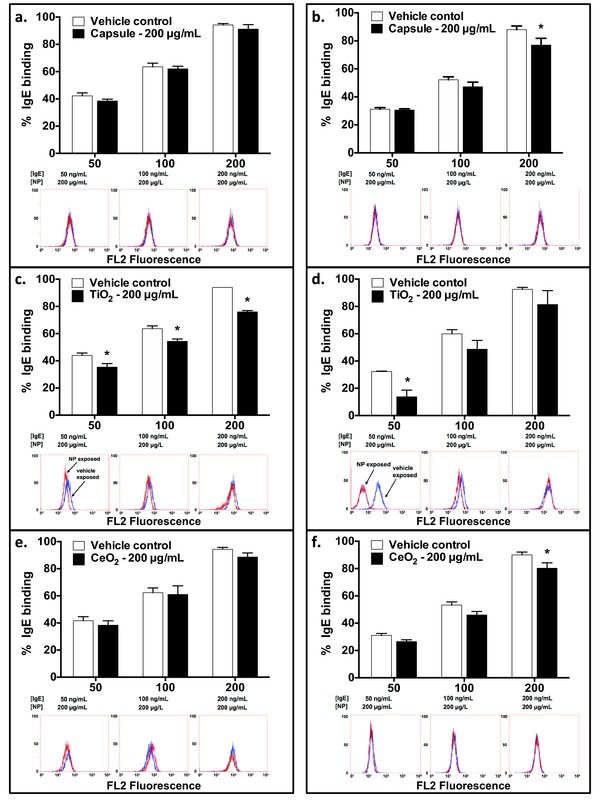
Relative percent expression of IgE bound on RBL‐2H3 cells. Panels a,c,e: RBL‐2H3 cells were pre‐exposed to 200 μg mL^−1^ PAA‐NPs ((a) capsule control; (c) TiO_2_; (e) CeO_2_) for 2 h followed by IgE sensitization (50, 100, 200 ng mL^−1^). Panels b,d,f: IgE (50, 100, 200 ng mL^−1^) was pre‐exposed to PAA‐NPs for 1 h prior to sensitization of unexposed RBL‐2H3 cells. Vehicle controls (cells or IgE; white bars in histogram; blue plots in flow cytometry outputs) were exposed to ddH_2_O in place of PAA‐NPs (black bars in histogram; red plots in flow cytometry outputs). Secondary goat‐anti mouse IgG antibody conjugated to phycoerythrin (PE) was added to sensitized cells and fluorescence measured by flow cytometry. Values from unexposed (NPs or IgE) controls were subtracted from all experimental values and data are presented normalized to positive controls. Data are means ± SEM *n* = 5–6 independent experiments. * denote significant differences (ANOVA, *p* < 0.05) between treatments at each IgE concentration.

IgE binding to RBL‐2H3 FcεRI was not significantly different than controls when RBL‐2H3 cells were pre‐exposed for 2 h to 200 μg mL^−1^ PAA‐Caps and PAA‐CeO_2_, followed by sensitization with IgE at 50, 100, 200 ng mL^−1^ (**Figure** 2a,e). However, IgE binding was significantly decreased (i.e., relative shift in MFI) (35.33 ± 2.67%, 54.33 ± 1.67%, and 76 ± 1.0%) at all IgE concentrations (50, 100, 200 ng mL^−1^, respectively) when pre‐exposed to 200 μg mL^−1^ PAA‐TiO_2_, compared to controls (44 ± 1.73%, 63.67 ± 2.03%, 94 ± 0.00%, respectively) (Figure 2c). No differences in receptor binding were observed for IgE concentrations below 50 ng mL^−1^ for all PAA‐NPs (data not shown).

### 2.4 Binding of IgE to FcεRI was Reduced when Pre‐exposed to PAA‐NPs

When pre‐exposed to a single concentration (200 μg mL^−1^) of PAA‐Cap or PAA‐CeO_2_ for 1 h, IgE antibody binding (at 200 ng mL^−1^) to FcεRI significantly decreased (PAA‐Cap: 77 ± 4.81%, PAA‐CeO_2_: 80.25 ± 3.99%, respectively) relative to paired controls (PAA‐Cap: 88 ± 2.59%, PAA‐CeO_2_: 90 ± 2.21%, respectively), while NP‐exposed IgE at 50 and 100 ng mL^−1^ were not different (Figure 2b,f). By contrast, pre‐exposure of IgE to a single concentration of PAA‐TiO_2_ (200 μg mL^−1^), resulted in significantly reduced IgE binding at the lowest IgE concentration (50 ng mL^−1^) (13.76 ± 4.88%), relative to unexposed paired control (32.29 ± 0.31%) (Figure 2d). Exposure of 100 and 200 ng mL^−1^ IgE to PAA‐TiO_2_ did not significantly change the percent of IgE bound to FcεRI. No differences in binding were observed for IgE concentrations below 50 ng mL^−1^ for all PAA‐NPs (data not shown).

When a single concentration of IgE (200 ng mL^−1^) was pre‐exposed for 1 h to 50, 100, and 200 μg mL^−1^ PAA‐Caps and PAA‐CeO_2_, no significant changes in relative IgE binding were observed (Supplementary Figure 3, Supporting Information). However, the IgE binding decreased in a dose‐dependent manner (68.33 ± 12.35%, 31.33 ± 3.84%, and 17 ± 0.58%, respectively), compared to unexposed control cells (100.3 ± 6.98%), when a single concentration of IgE (200 ng mL^−1^) was pre‐exposed for 1 h to 50, 100, 200 μg mL^−1^ PAA‐TiO_2_, (Supplementary Figure 3, Supporting Information). Heat denaturing of IgE (negative control) resulted in negligible IgE binding (3.0 ± 3.00%), indicating that fluorescence from secondary IgG‐PE antibody required functional IgE to be measured by flow cytometry.

### 2.5 MAPK ERK Phosphorylation was Reduced when Cells or IgE were Exposed to PAA‐NPs

**Figure 3:**
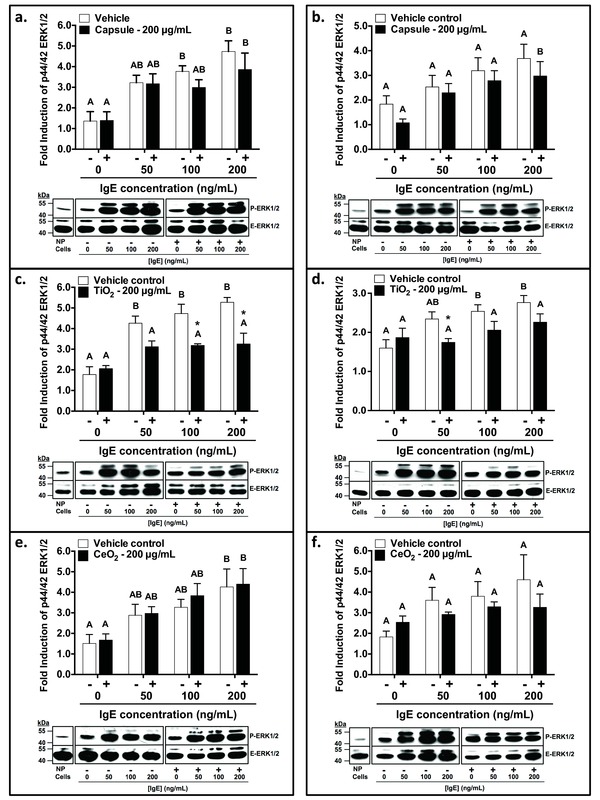
Relative percent induction of ERK1/2 p44/42 MAP Kinase (MAPK) pathway in RBL‐2H3 cells. Panels a,c,e: RBL‐2H3 cells were pre‐exposed to 200 μg mL^−1^ PAA‐NPs ((a) capsule control; (c) TiO_2_; (e) CeO_2_) for 2 h followed by IgE sensitization (50, 100, 200 ng mL^−1^). Panels b,d,f: IgE (50, 100, 200 ng mL^−1^) was pre‐exposed to 200 μg mL^−1^ PAA‐NPs for 1 h prior to sensitization of unexposed RBL‐2H3 cells. Vehicle controls (cells or IgE; −, white bars) were exposed to ddH_2_O in place of PAA‐NPs (+, black bars). RBL‐2H3 cells were activated with 0.1 × 10^−6^ m DNP‐HSA. Relative percent change in MAPK ERK phosphorylation was determined by correcting for the expression of endo‐ERK1/2 p44/42, then normalizing to unexposed negative control cells. Data are means ± SEM *n* = 4 independent experiments. Different upper‐case letters denote significant differences (one‐way ANOVA, *p* < 0.05) between IgE concentrations for each treatment group, followed by a pairwise Tukey multiple comparison test. * denote significant differences (two‐way ANOVA, *p* < 0.05) within a given IgE concentration, followed by a Bonferroni multiple comparison test.

Untreated control cells express relatively low levels of phosphorylated MAPK ERK. The addition of IgE at 50, 100 or 200 μg mL^−1^ induced dose‐dependent phosphorylation in vehicle‐exposed cells for both the NP‐exposed cell and NP‐exposed IgE experiments (**Figure** **3** 2). Cells that were not sensitized with IgE (0 ng mL^−1^) but were exposed to NPs (200 μg mL^−1^) also had increased phosphorylated MAPK ERK for PAA‐TiO_2_ and PAA‐CeO_2_ in both experimental approaches. This increase was less pronounced when the PAA‐Caps were used.

Cells that were pre‐exposed to PAA‐TiO_2_ for 2 h prior to sensitizing with IgE had significantly decreased phosphorylated MAPK ERK at both mid (100 ng mL^−1^) and high (200 ng mL^−1^) IgE concentrations (3.18 ± 0.08%, 3.25 ± 0.53%, respectively), relative to vehicle‐exposed cells at the same IgE concentrations (4.73 ± 0.45%, 5.27 ± 0.24%, respectively) (Figure 3c). IgE at 50 ng mL^−1^ also had reduced MAPK ERK phosphorylation (3.12 ± 0.28%), although not significantly different than paired vehicle control (4.26 ± 0.35%) (Figure 3c). By contrast, cells that were pre‐exposed to PAA‐CeO_2_ and PAA‐Caps for 2 h did not have significantly decreased phosphorylated MAPK ERK at each IgE concentration (50, 100, 200 ng mL^−1^) (CeO_2_: 2.97 ± 0.33%, 3.87 ± 0.59%, 4.40 ± 0.76%; Cap: 3.17 ± 0.48%, 2.99 ± 0.38%, 3.86 ± 0.80%, respectively) relative to vehicle‐exposed cells at the same IgE concentrations (50, 100, 200 ng mL^−1^) (CeO_2_: 2.88 ± 0.53%, 3.27 ± 0.39, 4.26 ± 0.88%; Cap: 3.22 ± 0.36%, 3.77 ± 0.27%, 4.73 ± 0.52%, respectively) (Figure 3a,e).

When cells were sensitized with IgE pre‐exposed to PAA‐TiO_2_, a significant decrease in phosphorylated MAPK ERK was observed for 50 ng mL^−1^ IgE (1.74 ± 0.10%), with a trend toward decreased phosphorylation for 100 ng mL^−1^ (2.057 ± 0.22%,) and 200 ng mL^−1^ (2.26 ± 0.21%) IgE, relative to vehicle‐exposed cells at the same IgE concentrations (2.34 ± 0.18%, 2.54 ± 0.17%, 2.76 ± 0.18%, respectively) (Figure 3d). Cells sensitized with PAA‐Cap‐exposed IgE also had decreased phosphorylated MAPK ERK at each IgE concentration (50, 100, 200 ng mL^−1^) (2.29 ± 0.38%, 2.78 ± 0.41%, 2.97 ± 0.59%, respectively), but this decrease was not significantly different than vehicle‐exposed IgE at the same IgE concentrations (2.53 ± 0.47%, 3.19 ± 0.53%, 3.68 ± 0.58%, respectively) (Figure 3b). Similarly, the phosphorylated MAPK ERK for cells sensitized with PAA‐CeO_2_‐exposed IgE was also slightly decreased (2.92 ± 0.10% 3.29 ± 0.24%, 3.26 ± 0.64%, respectively), relative to vehicle‐exposed IgE (3.60 ± 0.62%, 3.80 ± 0.70%, 4.60 ± 1.21%, respectively), at IgE concentrations of 50, 100, 200 μg mL^−1^, respectively, but this decrease was also not significantly different (Figure 3f).

### 2.6 RBL‐2H3 Cellular Degranulation was Reduced when Cells were Pre‐exposed to PAA‐NPs

**Figure 4:**
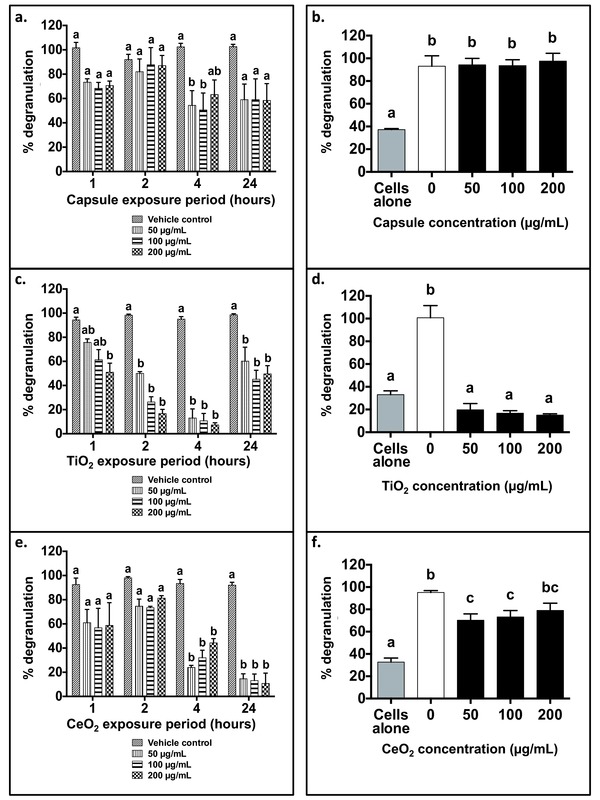
Relative percent degranulation (β‐hex release) of RBL‐2H3 cells. Panels a,c,e: RBL‐2H3 cells were pre‐exposed to 50, 100, 200 μg mL^−1^ PAA‐NPs ((a) capsule control; (c) TiO_2_; (e) CeO_2_) for 1, 2, 4 or 24 h followed by IgE sensitization (200 ng mL^−1^) for 1 h. Panels b,d,f: IgE (200 ng mL^−1^) was pre‐exposed to 50, 100, 200 μg mL^−1^ PAA‐NPs for 1 h prior to sensitization of unexposed RBL‐2H3 cells. Vehicle controls (cells or IgE) were exposed to ddH_2_O in place of PAA‐NPs. Degranulation assay (excitation/emission: 360 and 450 nm, respectively) reagents were added to experimental groups following exposures. Values from unexposed controls were subtracted from all experimental values and data are presented as normalized to positive controls (0.1 ng mL^−1^ DNP‐HSA). Data are means ± SEM *n* = 5–6 independent experiments. Different lower‐case letters denote significant differences (two‐way ANOVA, *p* < 0.05) within an RBL‐2H3 exposure period for each PAA‐NP (panels a,c,e), followed by a Bonferroni multiple comparison test. Different lower‐case letters denote significant differences (one‐way ANOVA, *p* < 0.05) between exposed IgE (panels b,d,f), followed by a pairwise Tukey multiple comparison test.

Degranulation decreased following PAA‐NP exposures to RBL‐2H3 cells, with the magnitude dependent on NP type, concentration and length of exposure (**Figure** 4). For example, at 1 h, 200 μg mL^−1^ PAA‐TiO_2_ significantly decreased RBL‐2H3 degranulation (51.1 ± 7.60%) (Figure 4c), relative to controls (94.4 ± 2.40%), however, exposures to PAA‐Caps and PAA‐CeO_2_ at the same concentrations did not (Figure 4a,e). After 4 h, degranulation levels decreased to 7.3 ± 1.77% of control (95.1 ± 2.20%) from exposures to 200 μg mL^−1^ PAA‐TiO_2_ (Figure 4c). However, after 24 h, the degranulation values for PAA‐TiO_2_ were comparable as those observed at 1 h. Similar dose‐ and time‐dependent reductions in degranulation were observed for PAA‐CeO_2_ over time (Figure 4e). However, unlike PAA‐TiO_2_, the reduction continued to decrease with longer PAA‐CeO_2_ exposure periods up to 24 h, and this occurred at doses that did not affect cellular viability. Degranulation also significantly decreased from exposure to PAA‐Cap, by approximately two fold for all tested doses at 4 h (50 μg mL^−1^: 54.3 ± 12.0%, 100 μg mL^−1^: 50.7 ± 14.0%, 200 μg mL^−1^: 63.4 ± 12.1%) (Figure 4a). After 24 h, degranulation remained inhibited. Finally, PAA‐ZnO and PAA‐Fe_2_O_3_‐exposed cells also had reduced degranulation values (Supplementary Figure 4, Supporting Information), despite also not eliciting a reduction in viability at any dose or exposure period. Exposures less than 10 μg mL^−1^ did not significantly affect degranulation for all NPs and exposure periods (data not shown).

### 2.7 RBL‐2H3 Degranulation was Abrogated when IgE was Pre‐exposed to PAA‐NPs

Degranulation was abrogated in RBL‐2H3 cells that were sensitized with IgE (200 ng mL^−1^) pre‐exposed to 50, 100, and 200 μg mL^−1^ PAA‐TiO_2_ (50 μg mL^−1^: 19.75 ± 5.45, 100 μg mL^−1^: 16.75 ± 2.18, and 200 μg mL^−1^: 15.00 ± 1.23%), when compared to cells sensitized with unexposed IgE (100.8 ± 10.73%), (Figure 4d). As well, PAA‐TiO_2_ lowered the relative release of β‐hex below unsensitized negative control cells (33 ± 3.48%). Cells sensitized with IgE pre‐exposed to PAA‐CeO_2_, also displayed significantly lower degranulation at each NP concentration (50 μg mL^−1^: 70.25 ± 5.66%, 100 μg mL^−1^: 73.25 ± 5.66% and 200 μg mL^−1^: 79.00 ± 6.55%), but to a lesser extent than PAA‐TiO_2_ (Figure 4f). On the contrary, cells sensitized IgE pre‐exposed to PAA‐Cap (50, 100, 2000 μg mL^−1^), did not have decreased degranulatory responses (50 μg mL^−1^: 94.25 ± 5.66%, 100 μg mL^−1^: 93.50 ± 5.24% and 200 μg mL^−1^: 97.50 ± 6.93%), relative to unexposed cells (93.00 ± 9.23%) (Figure 4b).

### 2.8 RBL‐2H3 Degranulation Recovers when PAA‐TiO_2_ is Removed from Exposure Solution

**Figure 5:**
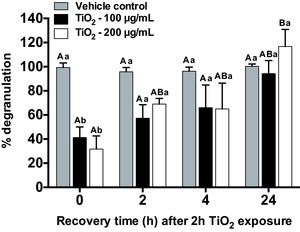
Relative percent degranulation (β‐hex release) of RBL‐2H3 cells exposed to 100 μg mL^−1^ or 200 μg mL^−1^ PAA‐TiO_2_ for 2 h. Exposure solutions were replaced with fresh cell media for 2, 4, and 24 h recovery periods, followed by sensitization with IgE (200 ng mL^−1^). Vehicle controls were exposed to ddH_2_O in place of PAA‐NPs. Degranulation assay (excitation/emission spectra: 360 and 450 nm, respectively) reagents were added to experimental groups following each recovery time point. Values from unexposed controls were subtracted from all experimental values and normalized to positive controls (0.1 ng mL^−1^ DNP‐HSA). Data are means ± SEM *n* = 5–6 independent experiments. Different lower case letter denote significant differences (two‐way ANOVA, *p* < 0.05) for each recovery duration period, followed by a Bonferroni multiple comparison test. Different upper‐case letters denote significant differences (one‐way ANOVA, *p* < 0.05) between recovery periods for each exposure concentration, followed by a pairwise Tukey multiple comparison test.

Despite a significant decrease in degranulation after a 2 h exposure to 100 and 200 μg mL^−1^ PAA‐TiO_2_ (41.33 ± 8.67% and 31.67 ± 10.93%, respectively), replacing PAA‐TiO_2_ with fresh MEM culture media resulted in a time‐dependent return to control degranulation levels for both tested doses over 24 h (**Figure** **5**). The 24 h time point also resulted in significantly more degranulation for the 200 μg mL^−1^ dose (116.8 ± 14.10%), relative to control cells (100.3 ± 1.97%).

## 3 Discussion

### 3.1 General Summary of Results

This study demonstrates that PAA‐NPs can affect the viability of mammalian granulocytes and these effects were dependent on the PAA‐NP core material, concentration, and duration of exposure. Our data also indicated that degranulation was suppressed, to varying extents, by all PAA‐NPs at sublethal doses, and especially by PAA‐TiO_2_. This may be due to decreased IgE binding by FcεRI and/or decreased activation of MAPK ERK when the RBL‐2H3 cells were exposed to PAA‐NPs. Together, these data suggest that PAA‐NP exposure significantly impacts the cellular degranulation response and by extension, granulocyte‐mediated innate immune responses.

### 3.2 Effects of PAA‐NPs on Cell Viability

We have demonstrated that PAA‐TiO_2_ significantly affected the viability of RBL‐2H3 cells (Supplementary Figure 2, Supporting Information), which was a similar response observed in primary goldfish (*Carassius auratus*) neutrophils.^22^ Although PAA‐NPs were more toxic to neutrophil viability than to RBL‐2H3 cells, similar trends associated with concentration, exposure duration, and particle type were observed. Results from previous studies show that PAA‐NPs have a stronger negative charge (ranging from −20 to −50 mV), smaller primary particle size (5–10 nm), higher dispersiveness and lower aggregation behaviors^10^, ^22^ than their unfunctionalized metal‐oxide counterparts in low ionic strength solutions.^23^ These physico‐chemical properties have been credited with lessening toxicity to fibroblast^3^ and human neutrophils^24^ that is typically observed when cells are exposed to unfunctionalized NPs using similar dosing (<100 μg mL^−1^) and exposure (<48 h) conditions.^3^

In particular, the strong negative charge of PAA‐NPs is believed to mitigate the negative effects on cell viability, as there would be less electrostatic interaction and disruption of negatively charged phosphatidylcholine and phosphatidylserine vesicles of the plasma membrane when exposed to cationic NPs.^25^ However, anionic PAA‐NPs could still mediate cell toxicity by interacting with blood proteins, creating protein coronas (i.e., the protein adsorption layer that forms around NPs^26^) that influence the fate and transport of NPs within circulation and into the cells of animals.

Surprisingly, cell viability was distinctly affected by each PAA‐NPs, despite having identical surface coatings. The most significant decreases were observed with PAA‐TiO_2_, which may be related to the smaller sized aggregates that form when in solution after 24 h when compared to the other PAA‐NPs.^22^ This would increase the number of single PAA‐TiO_2_ NPs in suspension and the available surface area to adversely interact with cells. Other considerations may include the amount of PAA polymer formed on the surface of the NP. During synthesis, the interaction between the inorganic metal ions and the PAA polymers is a charge‐dependent process. Thus, ZnO may attract more PAA than TiO_2_, for example, and form thicker polymer coats that could mask adverse core material‐mediated effects such as toxicity. The effects on viability were likely not related to the release of core metal ions as the dissolution of PAA‐NPs have not been demonstrated in ultrapure water,^10^ or in simulated biological solutions.^23^ As well, viability did not decrease over 24 h when RBL‐2H3 cells were exposed to Ti, Zn, Fe, and Ce ions (data not shown) at dialyzed concentrations previously determined for PAA‐NPs.^10^

### 3.3 Effects of PAA‐NPs on Cellular Degranulation

PAA‐NPs (PAA‐CeO_2_, PAA‐Fe_2_O_3_, PAA‐Caps) also significantly inhibited cellular degranulation at concentrations that did not affect viability at any PAA‐NP dose or exposure duration when RBL‐2H3 cells were pre‐exposed. The pattern of inhibition was also NP‐specific. For example, PAA‐TiO_2_ was the only PAA‐NP that exhibited a dose and time‐dependent effect when cells were exposed, while PAA‐ZnO, PAA‐Caps, and PAA‐Fe_2_O_3_ had more sustained inhibitory effects over 24 h. Exposure to PAA‐CeO_2_ resulted in a delayed effect, with inhibition occurring after 4 and 24 h. These differences are likely related to changes in their physico‐chemical properties over time, when in suspension.^22^ For example, over a 24 h period, discrete NP aggregates (>150 nm) formed for PAA‐CeO_2_ and PAA‐ZnO,^22^ signifying that NP surface areas that were available to interact with membrane receptors also decreased over time. We believe that this may have been responsible for the mitigation of the effect on degranulation in these materials. PAA‐TiO_2_ on the other hand was a highly monodispersed NP that had the smallest‐sized aggregates as measured by DLS (see Supplementary Table 1, Supporting Information). PAA‐TiO_2_ also demonstrated the most immediate effects on degranulation suggesting that the increased surface area to volume ratio available for PAA‐TiO_2_ NPs may have increased the number of individual particles that could interact with FcεRI, without inadvertently cross‐linking and activating receptor‐mediated degranulation, which has been previously shown with DNP‐coated gold NPs.^20^ With an estimated 3.0 × 10^5^ FcεRI per cell, the distance between adjacent receptors is approximately 32.6 nm.^20^ As such, larger NPs (>20 nm) that possess a bound cell‐activating ligand, such as DNP, could inadvertently increase degranulation via increased receptor oligomerization,^20^ while smaller NPs, such the PAA‐coated ones (<10 nm) used in this study, would be less likely to do so. Thus, PAA‐NP aggregation states likely play an important role in regulating RBL‐2H3 degranulation, which is influenced by core material properties, despite having identical surface functionalization. Further experimentation would be required to decipher the precise details of these interactions but our results support this notion.

Our findings are in agreement with other studies of innate granular cells following NP exposures to primary human mast cells,^27^ primary murine peritoneal mast cells,^28^ primary fish neutrophils,^29^ and RBL‐2H3 cells.^14^, ^20^ Conversely, other studies have reported that some NPs (bare TiO_2_) augment degranulation by directly activating l‐type voltage‐gated Ca^2+^ channels by stimulating the influx of extracellular Ca^2+^, triggering signaling cascades that activate histamine release.^18^ Bare TiO_2_ NPs can also act as an adjuvant in the generation of ovalbumin‐specific IgE and IgG antibodies,^15^ which in turn could sensitize mast cells to augment future allergic reactions.

### 3.4 Plausible Explanations for Suppressed Degranulation

There are reports that carbon‐based fullerenes suppress mast cell degranulation by scavenging free radicals, thereby reducing cellular reactive oxygen species (ROS) levels required to activate degranulation.^27^ It has also been proposed that C_60_ fullerenes inhibit degranulation in mast cells by competitively preventing granule release from SNARE‐bound secretory vesicles,^14^ whilst Maurer‐Jones et al.,^28^ have suggested that cell absorbed SiO_2_ NPs (25 nm) sufficiently interfere with cytoskeletal and membrane‐fusion machinery to prevent the exocytosis of cellular granules. We cannot discount the possibility that internalization of PAA‐NPs through membrane recycling events results in disruption of the degranulation responses. Internalization of NPs in immune cells has been shown to occur through clathrin and nonclathrin mediated endocytosis.^30^ However, given that pre‐exposing IgE to PAA‐NPs prior to sensitizing unexposed cells reduces membrane binding and degranulation, suggests that cell responses can also be inhibited at the plasma membrane level. Future studies will focus on the potential for internalization of these PAA‐NPs and the possibility that disruption of intracellular processes involved in degranulation also contribute to the observed inhibitory effects. In our study, we believe that suppression of the degranulation response occurs when receptor‐mediated events are obstructed. The size architecture of NPs has been reported to play a significant role in the activation or suppression of degranulation in mast cells where DNP‐coated gold NPs larger than 19.8 nm, cross‐linked FcεRI resulting in increased β‐hex secretion. However, when these NPs were less than 19.8 nm, cross‐linking did not occur and less β‐hex was released.^20^

Given that the primary particle diameters of the PAA‐NPs tested in this study are between 3 and 10 nm^10^, ^22^ and have high negative charge,^22^ we investigated the hypothesis that these smaller materials may still bind FcεRI and sterically obstruct IgE from properly attaching to the receptor and therefore inhibit the degranulatory response. Our data showed that cells pre‐exposed to PAA‐TiO_2_ had significant reductions in IgE binding, relative to control, suggesting that PAA‐TiO_2_ altered the attachment of IgE to FcεRI. These findings confirm the results of Huang et. al.,^20^ in demonstrating that NPs can interact directly with FcεRI and also extend these results by reporting that even smaller particles (3–10 nm) can inhibit cell effector functions.

### 3.5 Effects of PAA‐NPs on Cellular MAPK ERK Phosphorylation

In support of our findings, antigen‐induced phosphorylation of the receptor signaling target, MAPK ERK, was significantly reduced when cells were pre‐exposed to PAA‐NPs implying that alterations in IgE binding occurred. MAPK ERK are receptor signaling kinases that direct the regulation and activation of a diverse array of cellular processes, including proliferation, differentiation, and apoptosis, as well as several pro‐inflammatory functions (cytokine and eicosanoid production, phagocytosis, degranulation) in mast cells.^31^ MAPKs, such as ERK, are catalytically inactive at resting state, but when phosphorylated from upstream signaling pathways initiated from FcεRI subunits such as LYN, they become activated to regulate cell functions.^17^ Thus, our results support the hypothesis that partial reductions in MAPK ERK phosphorylation contribute to decreased RBL‐2H3 degranulation when RBL‐2H3 cells were pre‐exposed to PAA‐NPs.

Similarly, when IgE was pre‐exposed to PAA‐TiO_2_ and PAA‐CeO_2_, our results demonstrated that the efficacy of IgE to sensitize RBL‐2H3 cells and mediate degranulation via MAPK ERK signaling pathways was reduced. This was particularly true for PAA‐TiO_2_ exposures at lower IgE concentrations (50 ng mL^−1^), where a distinct separation in the binding of IgE for FcεRI was observed (Figure 2d). When IgE was first pre‐exposed to PAA‐TiO_2_, this treatment abrogated degranulation at all tested doses (Figure 4d) and also below control levels in non‐treated cells, indicating that PAA‐TiO_2_ also inhibited basal release of β‐hex. Interestingly, the magnitude of decreased MAPK ERK phosphorylation was similar regardless of whether RBL‐2H3 cells or IgE were pre‐exposed to PAA‐TiO_2_ suggesting that the modification of RBL‐2H3 degranulation by PAA‐NPs is likely mediated via the FcεRI.

Taken together, our results demonstrate that PAA‐TiO_2_‐exposed IgE reduced receptor binding capacity, thereby decreasing intracellular signaling (MAPK ERK) and ultimately reducing or abrogating the release of granular products (degranulation) that help promote pro‐inflammatory responses in vertebrates.

### 3.6 Implications of PAA‐NP Immune Modulation

The recovery of the degranulation response following the replacement of NP‐exposure media with NP‐free media demonstrates that the suppressive effects of PAA‐NPs are not permanent. PAA‐NPs were likely loosely bound to FcεRI and the dynamic interaction between the receptor, other proteins in solution, and the PAA‐NPs needs further investigation. We hypothesize that PAA‐NPs detached from the receptor following removal of NP‐exposure media, thereby allowing for IgE to bind FcεRI and the degranulatory process to be restored. This hypothesis is consistent with the concept of soft and hard protein coronas in which proteins that have lower affinities for NPs initially bind the NP, but are replaced by proteins with higher affinity for the NPs, creating the “hard” corona.^26^, ^32^ The implications could be significant if vital circulatory proteins, such as immunoglobulins (e.g., IgE, IgG), form part of the NP‐protein corona and are prevented from executing their biological roles. For example, we have previously demonstrated that lactate dehydrogenase (LDH) activity decreases when exposed to metal NPs by altering the conformational structure of the protein.^5^, ^7^ If animals are exposed to PAA‐based materials either directly as a therapeutic or inadvertently through environmental exposure, this may result in diminution of the functional activity of IgE mediated responses, and thereby impede the activation of immune function and alteration of their antimicrobial activity. Future research should focus on determining which blood proteins are bound by various NP formulations and whether this will impact the immune response of cells and organisms.

## 4 Conclusion

PAA‐NPs inhibited antigen‐induced RBL‐2H3 degranulation at sublethal doses. The decreased degranulation was mediated by affecting the attachment of IgE for its endogenous FcεRI, resulting in the diminished phosphorylation of MAPK ERK, and diminished RBL‐2H3 degranulation. Furthermore, each PAA‐NPs have been shown to distinctly modulate both viability and degranulation, despite having the same surface functionalization and primary particle sizes. These distinct differences in responses may be related to observed differences in their charge and agglomerating properties, particularly over time.^22^ These results further demonstrate that understanding physico‐chemical properties of NPs play important roles in determining effector functional responses of the innate immune system. Future studies should focus on the physico‐chemical properties of PAA‐NPs that modulate specific cellular toxicity and degranulation in immune cells.

## 5 Experimental Section

*Polymer‐Coated Metal‐Oxide Nanoparticle and Characterization Information*: The functionalized derivatives used in this study consisted of PAA‐coated metal oxide NPs, including TiO_2_ (rutile), ZnO, CeO_2_, Fe_2_O_3_ that were manufactured by Vive Crop Protection Inc. (formerly Vive Nano Inc.) (Toronto, ON, Canada) and were kindly donated for this study. Also included in the study were PAA nanocapsules (Caps) that do not have a metal core. These were used as a coating control for observed effects of the metal‐oxide NPs. Working concentrations were made from primary stock NPs (10 g L^−1^ in ddH_2_O) by diluting with ddH_2_O. All stocks were stored at 4 °C and protected from light. The methods used to synthesize are described by Felix et al.^10^ We had conducted extensive characterization of stock PAA‐NPs as previously reported.^6^, ^10^, ^22^ These included hydrodynamic diameter, polydispersity index, and zeta potential at 0 and 24 h for working stock solutions at concentrations between 10 and 200 μg mL^−1^ in ddH_2_O, plus transmission electron microscopy (TEM) images for each PAA‐NP. These data are reported previously,^22^ and is also presented in Supplementary Table 1. Additionally, primary particle size, pH, metal purity, and percent free metal released at 0.5 and 72 h as reported by Felix et al.^10^ is summarized in Supplementary Table 2, Supporting Information. The reported NP core sizes were measured between 3 and 9 nm using TEM (Supplementary Figure 5, Supporting Information) and the hydrodynamic sizes in suspension were approximately between 50 and 100 nm.^22^ Only Fe_2_O_3_ had larger aggregates at approximately 140 nm, likely due to their magnetic properties.^22^ Importantly, PAA‐NPs with all the cores remain highly monodispersed over 24 h and release only trace amounts of free metal ions over 72 h, with the majority of ions released in the first 30 min.^10^ Metal purity is above 98% for all PAA‐NP, aside from CeO_2_, with the remaining contaminating metal being the Na^+^ stabilizer used during the synthesis process. To determine the intrinsic absorbance and fluorescence of each PAA‐NP, absorbance and fluorescence spectrum measurements were performed and plotted (Supplementary Figure 1a, b, Supporting Information). Each PAA‐NP were diluted in ddH_2_O to 50 μg mL^−1^ and loaded into a quartz cuvette. An absorbance spectrum from 190 to 820 nm (Hewlett Packard 8452A diode array spectrophotometer) and a fluorescence emission spectrum up to 1100 nm (excitation 250 nm, Cary Eclipse photoluminescence spectrometer) were then recorded.

*RBL‐2H3 Cell Line*: The rat basophil RBL‐2H3 cell line was grown at 37 °C with 5% CO_2_ in culture media consisting of minimal essential medium (MEM) with Earle's balanced salt solution (Sigma‐Aldrich, Canada) supplemented with 1% 2 × 10^−3^ m l‐glutamine, 1% penicillin/streptomycin, and 10% heat‐inactivated FBS as described by Cortes et al.^21^ Cells were passed every third day by harvesting cells in an RBL‐2H3 harvest buffer (1.5 × 10^−3^ m EDTA, 135 × 10^−3^ m NaCl, 20 × 10^−3^ m HEPES, 5 × 10^−3^ m KCl, pH 7.4) at 37 °C with 5% CO_2_ for 10 min, followed by pipetting to detach cells from cell culture plate (BD Biosciences, Mississauga, Canada). Cells were seeded into new flasks at a sub‐cultivation ratio of 1:10.

*Examination of PAA‐NP Exposure on RBL‐2H3 Cell Viability Using Flow Cytometry*: Flow cytometry (Beckman Coulter Quanta SC, Mississauga, Canada) was used to measure the effects of NP exposure on cell viability, with propidium iodide (PI) used as a fluorescent marker for cell death. PI penetrates damaged membranes of necrotic and/or late apoptotic cells and intercalates with nucleic acids to enhance its fluorescence 20–30 times.^33^ The assay was performed by growing cells to confluence over 3 d in RBL‐2H3 culture media. Cells were harvested as described above and enumerated with Trypan Blue staining solution (Sigma‐Aldrich, Canada) on a hemocytometer to ensure cultures had >95% viable cells and to determine the number of cells in the culture.

Following enumeration, cells were resuspended in fresh culture media and seeded in 96‐well flat‐bottom culture plates (Corning Costar, USA) at 1.2 × 10^5^ cells per well for NP exposure periods less than 24 h. Cells were then incubated for 1 h at 37 °C to allow for cell attachment to wells. For viability experiments, RBL‐2H3 cells were exposed to one of five PAA‐NPs (TiO_2_, CeO_2_, ZnO, Fe_2_O_3_, Caps) at concentrations of 1, 10, 50, 100, 200 μg mL^−1^ for exposure periods of 1, 2, 4, and 24 h at 37 °C. Vehicle control cells received an equal volume of sterile double deionized (dd) water, while no additional solutions were provided to unexposed control cells.

Following each exposure, cell culture media was aspirated from wells and harvest buffer was added to each well. Cells were harvested as above and transferred to 1.5 mL centrifuge tubes and washed twice in phosphate buffered saline (PBS) by centrifugation at 400×*g* for 7 min to remove cell media and NPs. The cell pellets were gently disrupted and resuspended in a PBS/PI (100 μg mL^−1^) mixture and analyzed by flow cytometry (FL2 filter) for indications of cell death by monitoring for increases in PI fluorescence, and for changes in cell profile outputs, relative to unexposed control cells. Cell viability was determined by calculating the percentage of living cells that did not fluoresce for PI (i.e., not dead or dying) to the unexposed control population using the following equation [(percent viable of experimental treatment/percent viable of unexposed control cells) × 100].

*Experimental Approaches to Examine PAA‐NP Effects on the Degranulatory Signaling Cascade of Activated RBL‐2H3 Cells*: Figure 1 depicts the two experimental approaches used in this study to examine the effects of NPs on RBL‐2H3 degranulation. The first approach pre‐exposed RBL‐2H3 cells to PAA‐NPs, while the second approach pre‐exposed IgE antibodies to PAA‐NPs. For both approaches, measured endpoints included: cellular degranulation, IgE receptor binding, and MAPK signaling. The following sections describe the materials and methods for each measured endpoint.

*RBL‐2H3 Degranulation Experiments: Examination of PAA‐NP Exposures on RBL‐2H3 Degranulation*: Cellular activation and was assessed via the β‐hexosaminidase release assay.^19^ The assay was performed by seeding 5.0 × 10^4^ RBL‐2H3 cells per well, suspended in cell culture media, into a 96‐well flat‐bottom plate (Costar). Cells were then incubated for 1 h at 37 °C to allow for cell attachment, followed by triplicate dosing of each PAA‐NP (TiO_2_, CeO_2_, ZnO, Fe_2_O_3_, Caps) at concentrations of 10, 50, 100, 200 μg mL^−1^ for 1, 2, 4, and 24 h at 37 °C. Treatment groups for these experiments included: Vehicle control, which received an equal volume of sterile ddH_2_O; Positive control, which included a IgE‐sensitized and DNP‐HSA stimulated group, and received an equal volume of cell culture media; and, Negative control, which also received an equal volume of cell culture media alone. Following treatments, exposure media were removed by aspiration and treatment groups were washed in Tyrode's buffer (25 × 10^−3^ m HEPES, 140 × 10^−3^ m NaCl, 1.8 × 10^−3^ m CaCl_2_, 5.6 × 10^−3^ m d‐glucose, 12 × 10^−3^ m NaHCO_3_, 0.37 × 10^−3^ m NaH_2_PO_4_, MgCl_2_, BSA 0.1%, pH 7.4). 200 ng mL^−1^ of mouse anti‐DNP IgE mAb (Sigma‐Aldrich, Canada) was then added to PAA‐NP exposed, vehicle control and the positive control groups for 1 h at 37 °C, while Tyrode's buffer alone was added to negative control groups. Following incubation, all solutions were removed and cells were again washed with Tyrode's buffer, and Tyrode's buffer containing 0.1 ng mL^−1^ DNP‐HSA (Biosearch Technologies Inc. USA) was added to PAA‐NP exposed, vehicle control, and positive control groups, while Tyrode's buffer alone to negative control group. All groups were incubated for 1 h at 37 °C. Following incubation, the levels of β‐hex released by the RBL‐2H3 cells were determined by incubating the cellular supernatants with 2 × 10^−3^ m of 4‐methylumbelliferyl n‐acetyl‐β‐d‐glucosaminide as described by Naal.^19^ A Perkin‐Elmer microplate reader (360 nm excitation; 450 nm emission) analyzed sample fluorescence. Relative fluorescence units (RFU), signifying the cellular release of β‐hex, were represented as normalized values by setting positive control cells to 100%. The relative release for all samples was then calculated using the following equation [(RFU of experimental treatment—RFU of negative control)/RFU of IgE positive control cells—RFU of negative control) × 100].

*Examination of RBL‐2H3 Degranulatory Recovery*: RBL‐2H3 cells were exposed as described above to 100 and 200 μg mL^−1^ PAA‐TiO_2_ for 2 h. The PAA‐TiO_2_ exposure media was then replaced with fresh culture media and degranulation measured at 0, 2, 4, and 24 h post‐replacement, to test if the PAA‐NPs had a permanent or temporary effect on RBL‐2H3 degranulation. The degranulation assay was conducted as described above. For each recovery time point, cells that did not have the PAA‐TiO_2_ exposure media removed were measured for degranulation to ensure that the responses were consistent with the degranulation experiments described in Section 2.5.

*Examination of PAA‐NPs on IgE‐mediated Degranulatory Properties*: Degranulation was assessed on RBL‐2H3 cells using IgE antibodies that were first pre‐exposed to PAA‐TiO_2_, PAA‐CeO_2_, and PAA‐Caps. IgE antibodies (200 ng mL^−1^) were pre‐exposed on ice for 1 h with varying PAA‐NP concentrations (50, 100, 200 μg mL^−1^) prior to sensitizing cells for 1 h at 37 °C. An equal volume of sterile ddH_2_O was used as a vehicle control. The degranulation assay was then conducted as described above.

*IgE Antibody Binding Experiments: Examination of PAA‐NP Exposures on IgE Binding to the Surface of RBL‐2H3 Cells*: Three separate experiments were conducted to determine whether IgE receptor binding changed in the presence of PAA‐NPs. In the first experiment, RBL‐2H3 cells were harvested and enumerated as described above and seeded in 96‐well culture plates at densities of 1.0 × 10^5^ cells per well and allowed to adhere. Cells were then exposed for 2 h at 37 °C to PAA‐Caps, PAA‐TiO_2_ or PAA‐CeO_2_ at 200 μg mL^−1^, and paired to ddH_2_O as vehicle controls. Following exposure, cells were washed with PBS and sensitized with IgE suspended in MEM cell culture media at five concentrations (12.5, 25, 50 100, 200 ng mL^−1^), or with MEM alone as a negative control. Cells were then incubated for 1 h at 37 °C. Following sensitization, cells were washed with ice‐cold PBS to remove unbound IgE, and 1.0 μg of phycoerythrin (PE)‐ conjugated secondary antibody (goat anti‐mouse IgG (H+L) —PE, Sigma‐Aldrich, Canada) was added to each treatment group and incubated on ice, in the dark for 30 min. An IgG secondary alone control group received 1.0 μg IgG‐PE antibody. Following incubation, cells were washed with PBS and harvested as described above and resuspended in Tyrodes buffer for analysis of PE fluorescence using flow cytometry. Mean fluorescence intensity (MFI) values were assigned to each treatment group to quantify differences.

The second and third experiments tested the binding of IgE to the FcεRI with and without prior exposure to PAA‐NPs. The second experiment exposed a single IgE concentration (200 ng mL^−1^) for 1 h to PAA‐TiO_2_, PAA‐CeO_2_, and PAA‐Cap at three concentrations (50, 100, 200 μg mL^−1^), to determine whether IgE binding would change with increasing concentrations of PAA‐NPs. An equal volume of ddH_2_O was used as a vehicle control and denatured IgE (90 °C for 20 min) was used as a negative control. Conversely, the third experiment exposed varying IgE concentrations (12.5, 25, 50, 100, 200 ng mL^−1^) to a single concentration (200 μg mL^−1^) of PAA‐TiO_2_, PAA‐CeO_2_, and PAA‐Cap, or ddH_2_O (vehicle control) to determine the effects on IgE binding at lower sensitizing IgE concentrations, relative to unexposed IgE. For these experiments, RBL‐2H3 cells were seeded as described above and sensitized with one of the PAA‐NP‐exposed IgE treatments for 1 h at 37 °C. Following sensitization, cells were washed with ice‐cold PBS buffer to remove unbound IgE, and 1.0 μg of PE‐conjugated IgG secondary antibody was added to each treatment group and incubated on ice, in the dark for 30 min. IgG secondary alone control groups received 1.0 μg IgG‐PE. Following incubation, PE fluorescence was analyzed using flow cytometry as described above.

*Assessment of PAA‐NP Exposures on RBL‐2H3 MAPK ERK1(p44)/ERK2(p42) Phosphorylation*: RBL‐2H3 MAPK ERK was used as a signal transduction biomarker in two experiments to assess changes in cell activation when exposed to PAA‐NPs. In the first experiment, cells were seeded at 5.0 × 10^5^ RBL‐2H3 cells per well into a flat‐bottom 6‐well plates (Corning Costar, USA) and incubated for 2 d at 37 °C in culture media to allow for confluent growth (≈1.5 × 10^6^ cells per well). Cells were then incubated with or without PAA‐TiO_2_, PAA‐CeO_2_ or PAA‐Cap at 200 μg mL^−1^ in culture media for 2 h at 37 °C. An equal volume of sterile ddH_2_O was used for vehicle controls. Negative control cells were left undisturbed. Treatments were then removed and cells were washed twice in PBS prior to sensitization for 1 h in culture media to 50, 100 or 200 ng mL^−1^ IgE for both NP‐exposed and vehicle control cells. Negative control cells did not receive IgE. DNP‐HSA (0.1 ng mL^−1^) was then added to IgE‐sensitized cells for 8 minutes to cross‐link the receptors and activate the cells. The second experiment was designed identically as above, however IgE antibodies (200 ng mL^−1^), instead of cells, were pre‐exposed with or without PAA‐TiO_2_, PAA‐CeO_2_, or to PAA‐Caps (200 μg mL^−1^) for 1 h and then used to sensitize the cells. For vehicle control treatments, IgE were pre‐exposed to an equal small volume of sterile ddH_2_O. In both experiments, cells activated with DNP‐HSA were washed twice with PBS and lysed with 200 μL of ice‐cold lysis buffer (1% Triton X‐100, 150 × 10^−3^ m NaCl, 50 × 10^−3^ m Tris, phosphatase, and protease inhibitors (Roche from Molecular Biology Services Unit, pH 8.0) using mechanical disruption. Cell lystates were then prepared for Western blotting techniques by diluting 1:1 with 2× Laemmli reducing buffer (0.5 m Tris, 10% glycerol, 10% SDS. 1% bromophenol blue, 5% β‐mercaptoethanol), boiled at 95 °C for 10 min, and then separated on a SDS‐PAGE gel (10% acrylamide, 10% SDS, 1.5 m Tris separating buffer). Proteins were then transferred to nitrocellulose membranes for 1 h at 100 V and blotted with anti‐ERK1(p44)/ERK2(p42) MAPK (L34F12) and anti‐phospho‐p44/p42 MAPK (Erk1/2) XP mouse mAb purchased from Cell Signaling Technologies (USA) at a final dilution of 1:4000 (v/v). Immunoreactive protein bands were detected using a goat anti‐rabbit IgG (H+L) HRP‐conjugated pAb (Bio‐Rad) at a final dilution of 1:5000 (v/v) and visualized on authoradiography film (GE Healthcare) using the SuperSignal West Pico Chemiluminescent Substrate kit (Pierce Biotechnology). Analysis of band intensity was performed using ImageJ v1.47, which was downloaded from http://rsbweb.nih.gov/ij/download.html.

*Statistics*: Statistical analyses were performed using the GraphPad 6.0 statistical software program. To investigate effects of NPs on cell viability, IgE receptor binding, and degranulation from NP‐exposed cells, a two‐way analysis of variance (ANOVA) with a Bonferroni multiple comparison test was performed. To investigate effects of NPs on cellular MAPK ERK phosphorylation and degranulation from NP‐exposed IgE, a one‐way ANOVA with a pairwise Tukey multiple comparison test was performed. A probability of *p* < 0.05 was considered significant. Data values are presented as mean ± standard error on the mean (SEM).

## Supporting information

As a service to our authors and readers, this journal provides supporting information supplied by the authors. Such materials are peer reviewed and may be re‐organized for online delivery, but are not copy‐edited or typeset. Technical support issues arising from supporting information (other than missing files) should be addressed to the authors.

Supplementary
