# Supplementary material for: Polymer‐Coated Metal‐Oxide Nanoparticles Inhibit IgE Receptor Binding, Cellular Signaling, and Degranulation in a Mast Cell‐like Cell Line
Source: Adv Sci (Weinh). 2015 Jul 14;2(11):1500104. doi: 10.1002/advs.201500104 (PMC5115347; doi:10.1002/advs.201500104)
Supplement: Supplementary file 1 — Supplementary [file ADVS-2-0f-s001.pdf]

Copyright WILEY-VCH Verlag GmbH & Co. KGaA, 69469 Weinheim, Germany, 2015.

ADVANCED SCIENCE
Open Access

Supporting Information

for *Adv. Sci.*, DOI: 10.1002/advs.201500104

Polymer-Coated Metal-Oxide Nanoparticles Inhibit IgE Receptor Binding, Cellular Signaling, and Degranulation in a Mast Cell-like Cell Line

*Van A. Ortega, James D. Ede, David Boyle, James L. Stafford, and Greg G. Goss**

Supplementary Figure 1: Spectral measurements of optical characteristics of PAA-NPs Absorbance (a) and fluorescence (excited at 250 nm) (b) characteristics of PAA-NPs (TiO_2, CeO_2, Fe_2O_3, ZnO, Cap) at 50 μg/mL.

Supplementary Figure 2: Relative percent viability of RBL-2H3 cells exposed to PAA-NPs. $5x10^5$ cells per well were exposed to 50, 100 or 200 μg/mL PAA-TiO_2 (a), PAA-CeO_2 (b), PAA-ZnO (c), PAA-Fe_2O_3 (d), PAA-Caps (e) for 1, 2, 4 or 24 h. Vehicle controls were exposed to ddH_2O in place of PAA-NPs. Following exposures, cells were analyzed by flow cytometry for viability using propidium iodide (PI) as a marker for cell death. PI fluorescence in NP-exposed treatment groups were normalized to unexposed control cells. Data are means ± SEM n = 5-7 independent experiments. Different lower-case letters denote significant differences (Two-way ANOVA, $p < 0.05$) within an exposure period for each PAA-NP, followed by a Bonferroni multiple comparison test.

Supplementary Figure 3: Relative percent expression of IgE bound on RBL-2H3 cells. IgE (200 ng/ml) was pre-exposed to 50, 100, 200 μg/mL PAA-TiO_2, PAA-CeO_2 and PAA-Cap for 1 h prior to sensitization of unexposed RBL-2H3 cells, followed by staining with a goat anti-mouse IgG antibody conjugated to phycoerythrin (PE). Vehicle controls (0 μg/mL) were exposed to ddH_2O in place of PAA-NPs. Heat-denatured IgE antibodies were used as a negative control. Fluorescence was measured by flow cytometry (representative PAA-TiO_2 profile displayed). Values were normalized to positive IgE control. Data are means ± SEM n = 4 independent experiments. Different lower-case letters denote significant differences (one-way ANOVA, $p < 0.05$) in IgE binding between NP concentrations for each PAA-NP, followed by pairwise Tukey multiple comparison test.

Supplementary Figure 4: Relative percent degranulation (β-hex release) of RBL-2H3 cells exposed to 50, 100, 200 μg/mL PAA-ZnO (a) or PAA-Fe_2O_3 (b) for 1, 2, 4 or 24 h followed by IgE sensitization (200 ng/mL) for 1 h. Vehicle controls (cells or IgE) were exposed to ddH_2O in place of PAA-NPs. Degranulation assay (excitation/emission: 360 and 450 nm, respectively) reagents were added to experimental groups following exposures. Values from unexposed controls were subtracted from all experimental values and data are presented as normalized to positive controls (0.1 ng/mL DNP-HSA). Data are means ± SEM n = 5-6 independent experiments. Different lower-case letters denote significant differences (two-way ANOVA, $p < 0.05$) within an exposure period for each PAA-NP, followed by a Bonferroni multiple comparison test.

Supplementary Figure 5: Transmission electron micrographs (JEOL-2010 (LaB6 filament) electron microscope with an accelerating voltage of 200 kV) of Vive Crop Protection polyacrylic acid (PAA) functionalized nanoparticles (a) PAA-TiO_2, (b) PAA-Fe_2O_3, (c) PAA-CeO_2 and (d) PAA-ZnO showing sizes ranging between approximately 3 to 9 nm. Images are summarized from (Ortega et al., 2013).

Supplementary Table 1: Dynamic light scattering results showing mean hydrodynamic diameter (nm), zeta potential (mV) and polydispersity index of 200 ug/l Vive Crop Protection polyacrylic acid (PAA) functionalized CeO_2, TiO_2, Fe_2O_3, ZnO and Caps suspended in ultrapure H_2O. Data is summarized from Ortega et al., 2013. For full characterization, see associated reference.

Supplementary Table 2: Primary particle size (nm), pH, metal purity (%) and percent free metal dialyzed following 30 min and 72 h of dialysis of Vive Crop Protection polyacrylic acid (PAA) functionalized CeO_2, TiO_2, Fe_2O_3, ZnO, and PAA-Caps suspended in ultrapure H_2O. Data is summarized from Felix et al., 2013.

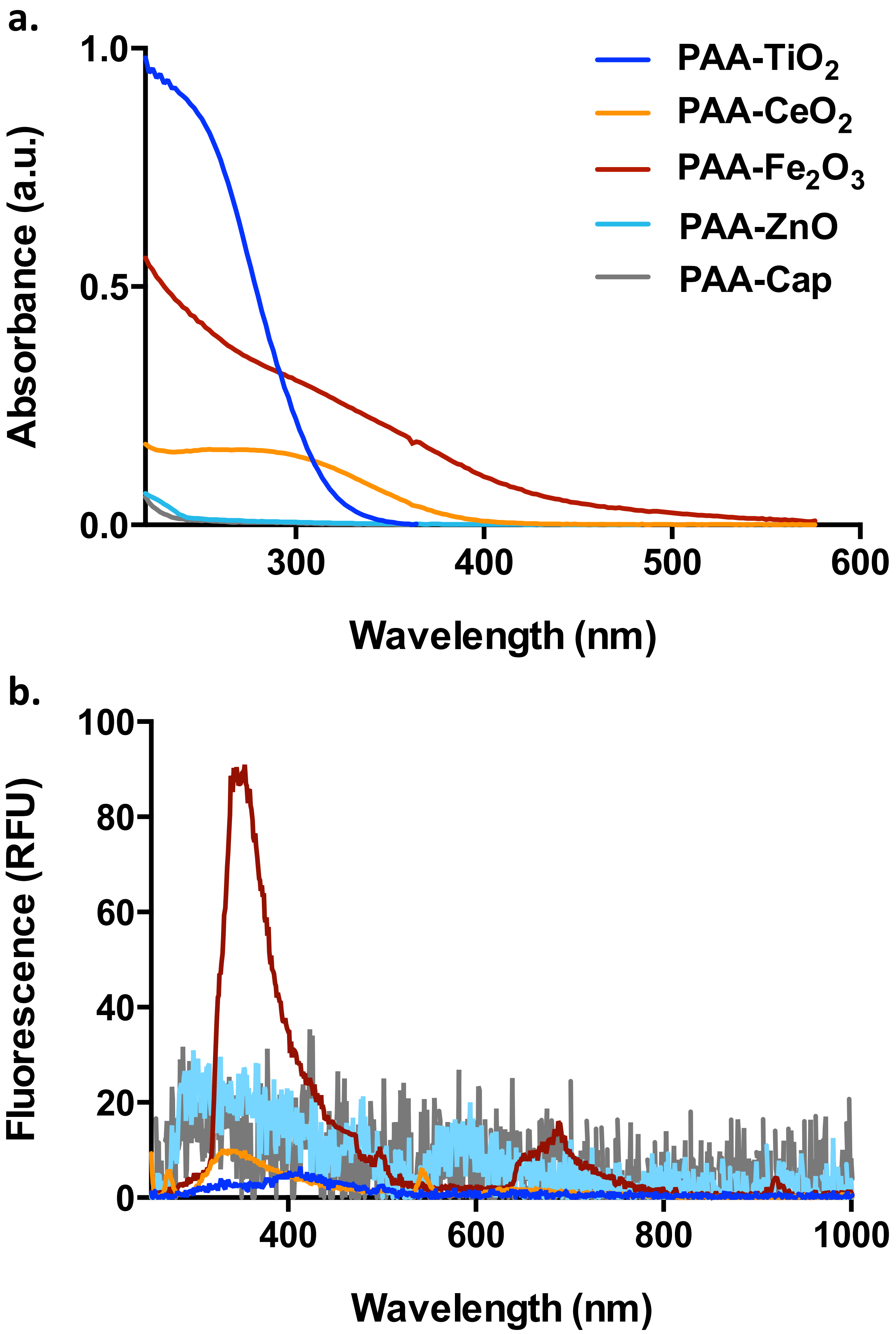

Supplementary Figure 1

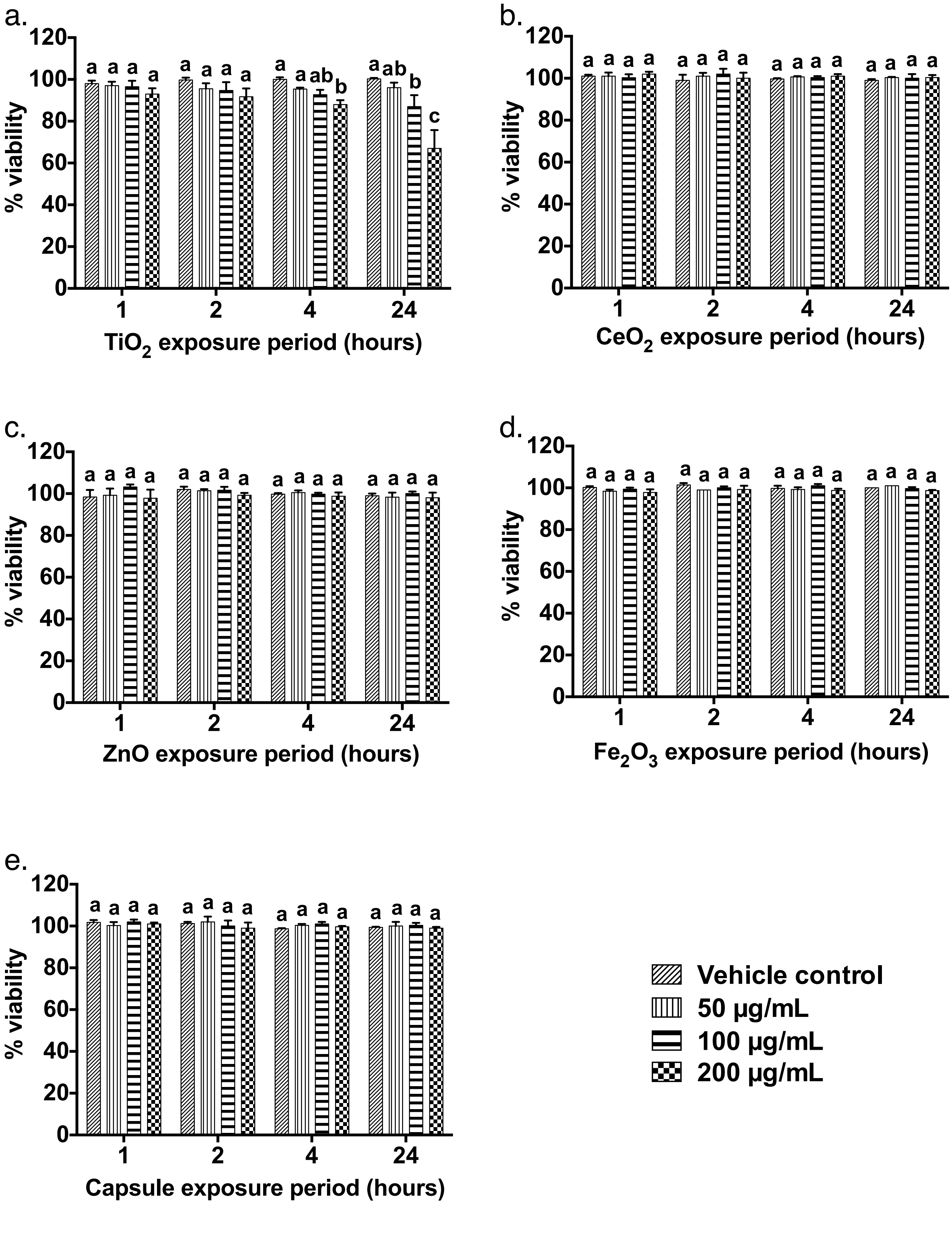

Supplementary Figure 2

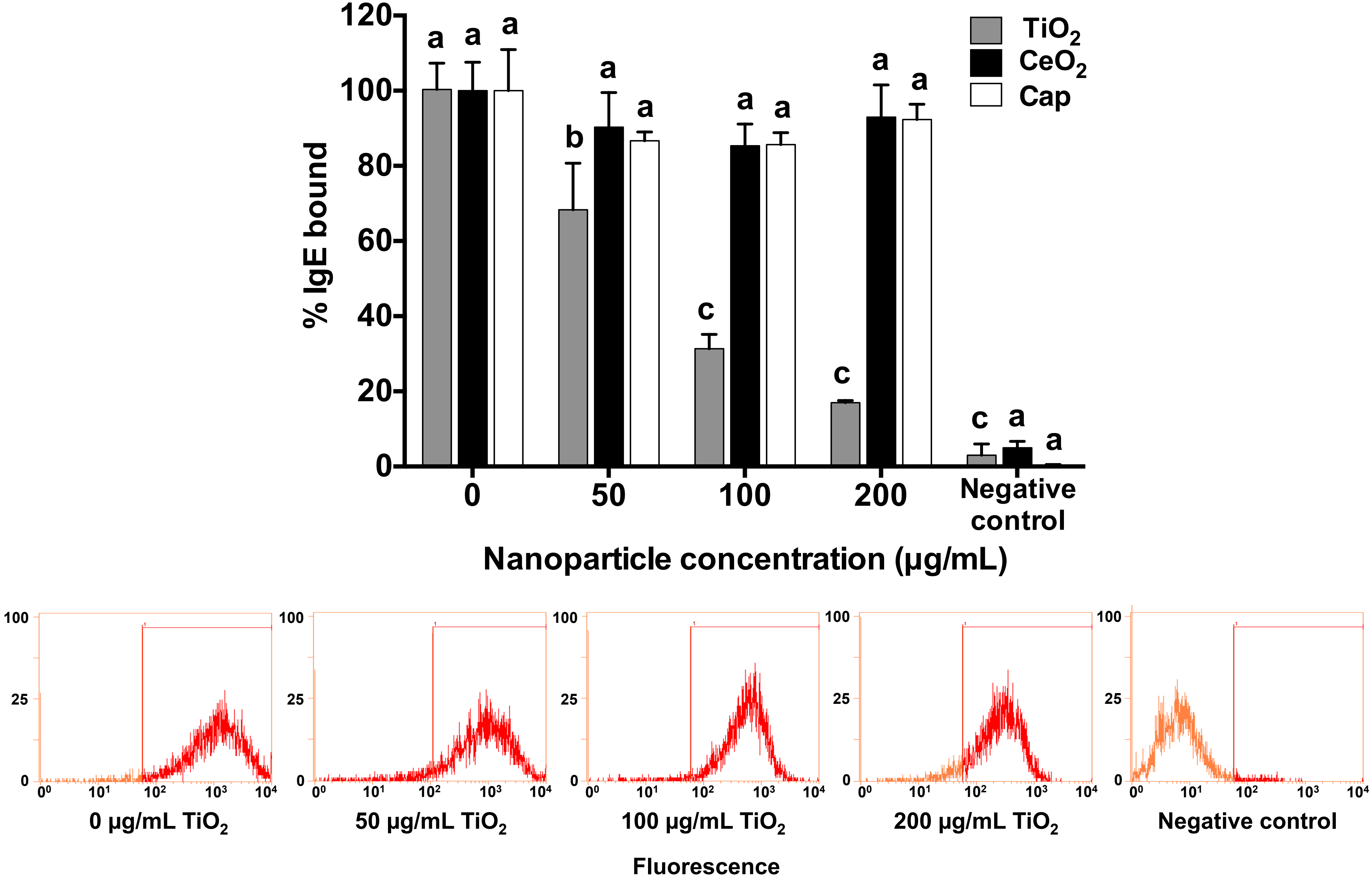

Supplementary Figure 3

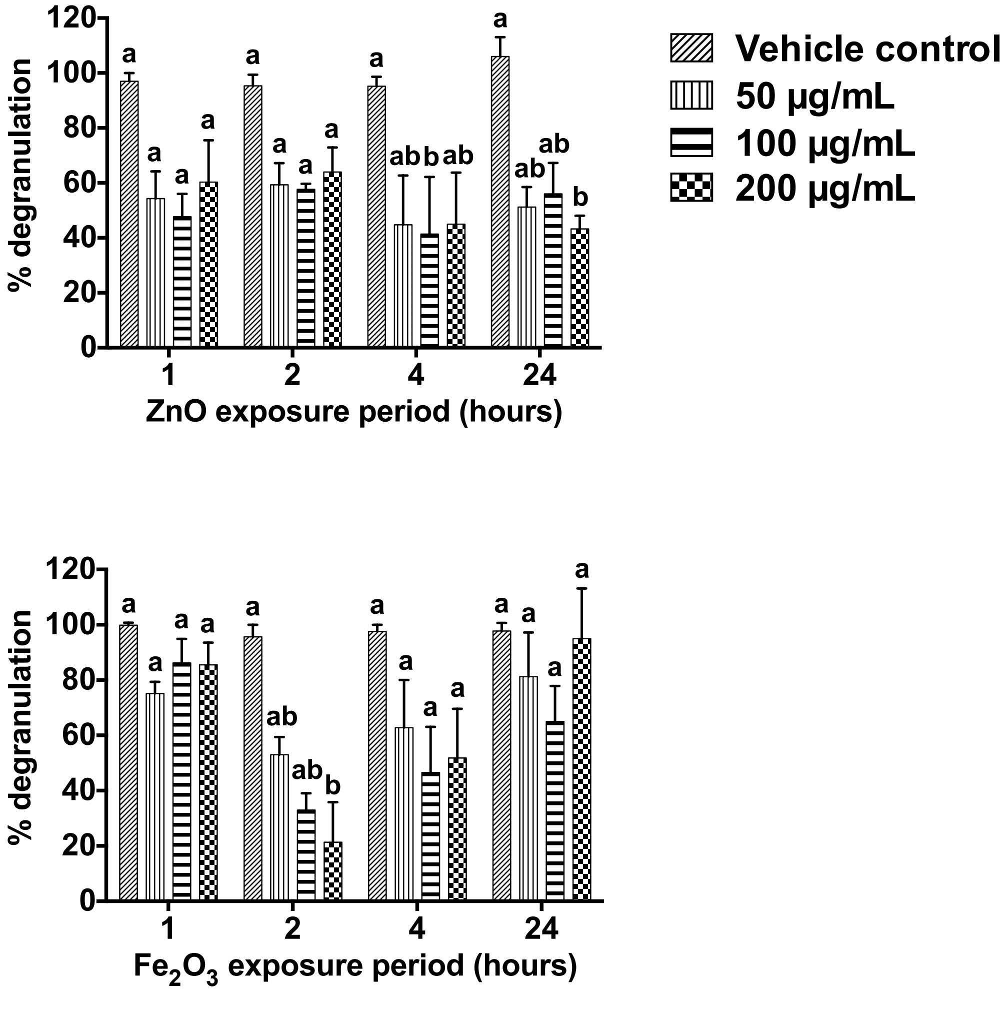

Supplementary Figure 4

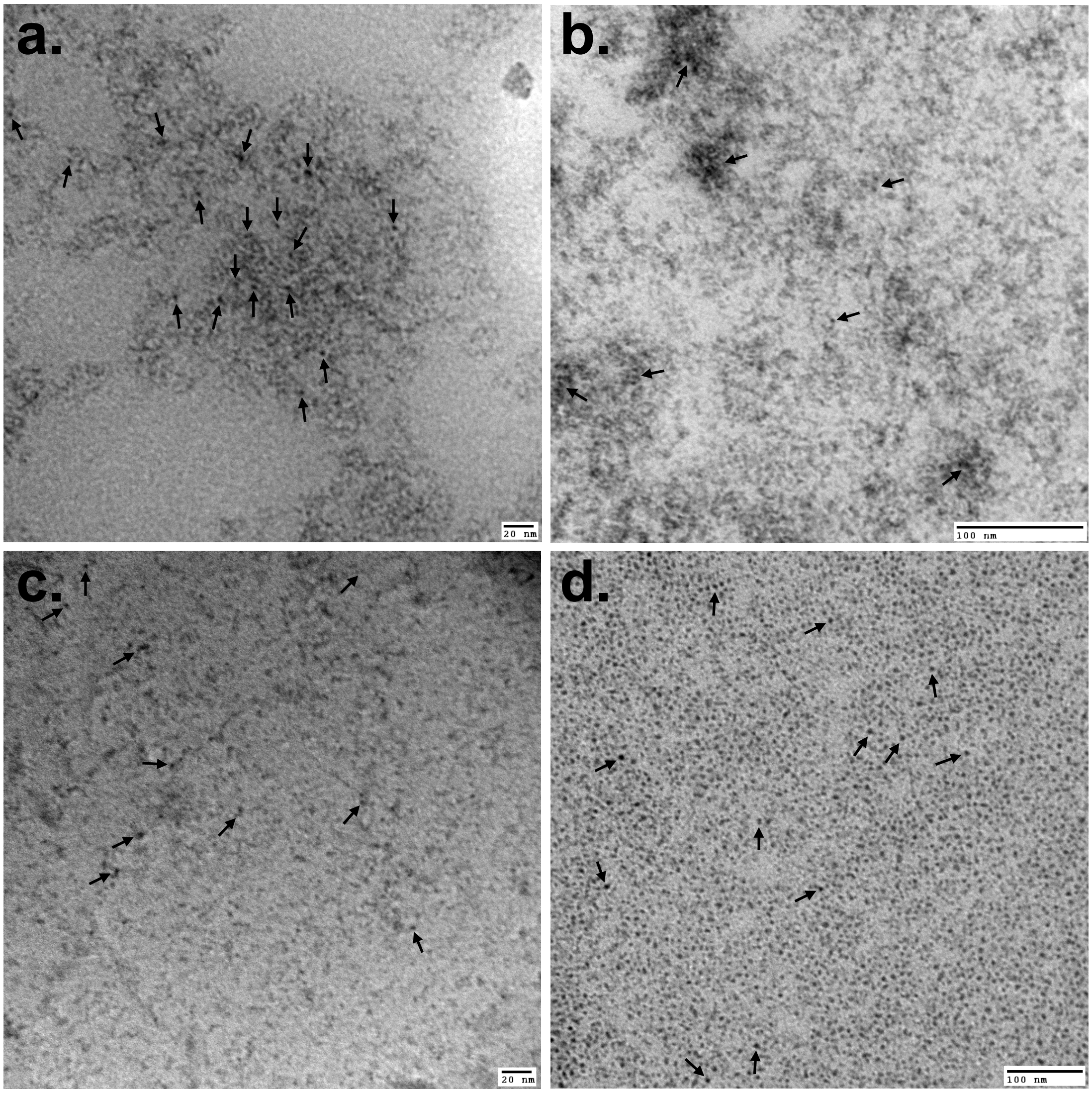

Supplementary Figure 5

Supplementary Table 1: Dynamic light scattering results showing mean hydrodynamic diameter (nm), zeta potential (mV) and polydispersity index of 200 μg/l Vive Crop Protection polyacrylic acid (PAA) functionalized CeO_2, TiO_2, Fe_2O_3, ZnO and Caps suspended in ultrapure H_2O. Data is summarized from Ortega et al. 2014. For full characterization, see associated reference.

**DLS Parameter**	**Nanoparticle**				
	**PAA-CeO_2**	**PAA-TiO_2**	**PAA-Fe_2O_3**	**PAA-ZnO**	**PAA-Caps**
HD (nm)	31	31	147	64	-
PDI	0.33	0.31	0.2	0.62	-
ZP (mV)	-41	-32	-52	-52	-30

Notes:
HD: Hydrodynamic diameter
PDI: Polydispersity Index
ZP: Zeta Potential

Supplementary Table 2: Primary particle size (nm), pH, metal purity (%) and percent free metal dialyzed following 30 min and 72 h of dialysis of Vive Crop Protection polyacrylic acid (PAA) functionalized CeO_2, TiO_2, Fe_2O_3, ZnO, and PAA-Caps suspended in ultrapure H_2O. Data is summarized from Felix et al. 2013.

**Nanoparticle**	**Primary particle size (nm)**[a]	**pH**	**Purity**[b]	**Percent free metal dialyzed after 0.5 h (%)**	**Percent free metal dialyzed between 0.5 - 72 h (%)**
PAA-CeO_2	3-9	8.9	88.0	0.73	0.015
PAA-TiO_2	3-9	7.0	98.0	1.07	0.016
PAA-Fe_2O_3	4.5	9.6	97.8	0.17	0.07
PAA-ZnO	3-9	8.9	99.7	0.002	ND
PAA-Caps	3-9	8.5	-	0.006	ND

Notes:

[a]Exclusive of PAA coating

[b]Purity is exclusive of Na^+ stabilizer and PAA coating.

Metal purity not measured for PAA-Caps due to the absence of a metal core.
